# 

**Published:** 1892-12

**Authors:** 


ESTABLISHED 1855. SUBSCRIPTION, $2.00.

### ATLANTA MEDICAL AND SURGICAL JOURNAL.

OLD SERIES, VOL. XXVI.
NEW SERIES, VOL. IX. } ATLANTA, GA., DECEMBER, 1892. NO. 10.

LUTHER B. GRANDY, M. D., MANAGING EDITOR.

M. B. HUTCHINS, M. D. BUSINESS MANAGER.

